# Induced abortion and future use of IVF treatment; A nationwide register study

**DOI:** 10.1371/journal.pone.0225162

**Published:** 2019-11-14

**Authors:** Jaana Männistö, Maarit Mentula, Aini Bloigu, Mika Gissler, Oskari Heikinheimo, Maarit Niinimäki

**Affiliations:** 1 Department of Obstetrics and Gynecology, PEDEGO Research Unit, Medical Research Center Oulu, University Hospital of Oulu and University of Oulu, Oulu, Finland; 2 Department of Obstetrics and Gynecology, University of Helsinki, and Helsinki University Hospital, Helsinki, Finland; 3 Finnish Institute for Health and Welfare, Helsinki, Finland; 4 Karolinska Institutet, Department of Neurobiology, Care Sciences and Society, Stockholm, Sweden; Johns Hopkins University Bloomberg School of Public Health, UNITED STATES

## Abstract

**Objective:**

In this nationwide study we assessed the use and factors associated with future *in vitro* fertilization (IVF) treatment after induced abortion.

**Materials and methods:**

The study population was collected by means of record linkage between Finnish national registers. All women who underwent induced abortion between 2000 and 2009 in Finland were identified through the Register of Induced Abortions (n = 88 522). The study group consisted of women who underwent induced abortion and subsequently had an IVF treatment (n = 379); the comparison group were all women who had a spontaneous pregnancy and delivery 12–24 months after the index abortion (n = 7434).

Demographic characteristics at the time of index abortion, and factors associated with the abortion (gestational age at abortion, indication and method of abortion, complications after abortion) were compared between the study groups. Logistic regression was used to assess whether some of the demographic characteristics or abortion associated factors increased the use of IVF treatment in the future.

**Results:**

The proportion of women with IVF treatment after induced abortion in the whole cohort was 0.4%. Women needing IVF treatment were older, of a higher socioeconomic status, and had fewer previous induced abortions and deliveries compared to women in the comparison group. No statistically significant differences were observed in the gestational age (≤ 12 weeks or >12 weeks of gestation) at abortion, method or complications of abortion. In multivariable analysis higher age increased, and history of previous deliveries or one or two abortions decreased the use of IVF.

**Conclusions:**

Infertility necessitating the use of IVF treatment after induced abortion is uncommon. The factors associated with use of IVF after abortion are those generally recognized as risk factors of infertility. Abortion-related outcomes are not associated with an increased need of future IVF-treatment.

## Introduction

Concerns about induced abortion jeopardizing future fertility have prevailed, even though studies from the 1980s and 1990s concluded that legal abortion has no adverse effects on future fertility [1–3]. The possible mechanisms how abortion could affect the fertility are likely to involve abortion affecting the fallopian tubes or endometrium due to infection, or mechanical trauma leading to infertility and future IVF treatments. However, there are no studies proving this theory.

Approximately half of women undergoing induced abortion have no previous births preceding abortion [4, 5] and the question of whether there are abortion-related factors, such as gestational age at abortion, method and complications of abortion, which may affect future fertility is important. The prevalence of infertility lasting for at least one year has been estimated to be approximately 15% in high-income countries [6]. In Finland, the rate of *in vitro* fertilization (IVF) treatment use among fertile aged women (15–49 years) was 7.5 per 1000 women in 2016, and altogether 6.5% of all children were born following assisted fertility treatments [5].

The procedure of induced abortion has changed dramatically during the past 20 years and surgical method has been replaced by a medication method in a growing number of countries. In Finland, the medication abortion with mifepristone and misoprostol was introduced in 2000 and the use of medication method has expanded since then: 97% of terminations were performed medically in 2017 [5]. The same upward trend can be seen in other countries, such as the United Kingdom and Norway [4, 7]. However, no studies on abortion and future fertility have been conducted since this change in abortion practice.

In the present study, we assessed the prevalence and the factors associated with future use of *in vitro* fertilization (IVF) treatment after induced abortion, and whether there are some abortion-related factors, such as gestational age at abortion, indication of abortion, method of abortion, or complications after abortion, associated with the use of these treatments. We compared women who suffered infertility treated by means of IVF after an abortion to women who had a spontaneous pregnancy and delivery within 12–24 months after the index abortion.

## Material and methods

In Finland, induced abortions are performed by healthcare professionals according to national law and guidelines [8]. Abortion is regulated by the legislation and requires an indication [9]. Indications include age less than 17 years or above 40 years at conception, having delivered at least four children, social circumstances, pregnancy being a risk for the woman’s life or health, suspected or confirmed anomaly or illness of the fetus or mother’s and/or father’s incapability to take care of the child, rape and incest or other reasons mentioned in the penal law. All abortions are to be registered to the Finnish Register of Induced Abortions maintained by the Finnish Institute of Health and Welfare [5]. The same institution maintains the Medical Birth Register, where all live births and stillbirths exceeding 22 gestational weeks or 500 grams in fetal weight are registered since 1987.

IVF treatments are provided in specialized public and private clinics, and usually include use of specific medications. Women below 40 years of age are entitled to low-cost IVF treatment provided by the public health care. In private clinics couples have to pay for the treatment. The medication prescriptions are reimbursed and filed in the Drug Reimbursement Register maintained by the Social Insurance Institution. It also partly reimburses the costs of treatments in private healthcare, including IVF treatments. These reimbursements are registered to the Procedure Reimbursement Register.

Furthermore, all Finnish hospitals provide information on diagnosis (International Classification of Diseases, ICD-10) [10] and procedures (NOMESCO Classification of Surgical Procedures) [11] of treatment episodes to the Hospital Discharge Register on all inpatient (all hospitals) and outpatient treatment visits (public hospitals).

**Fig 1:**
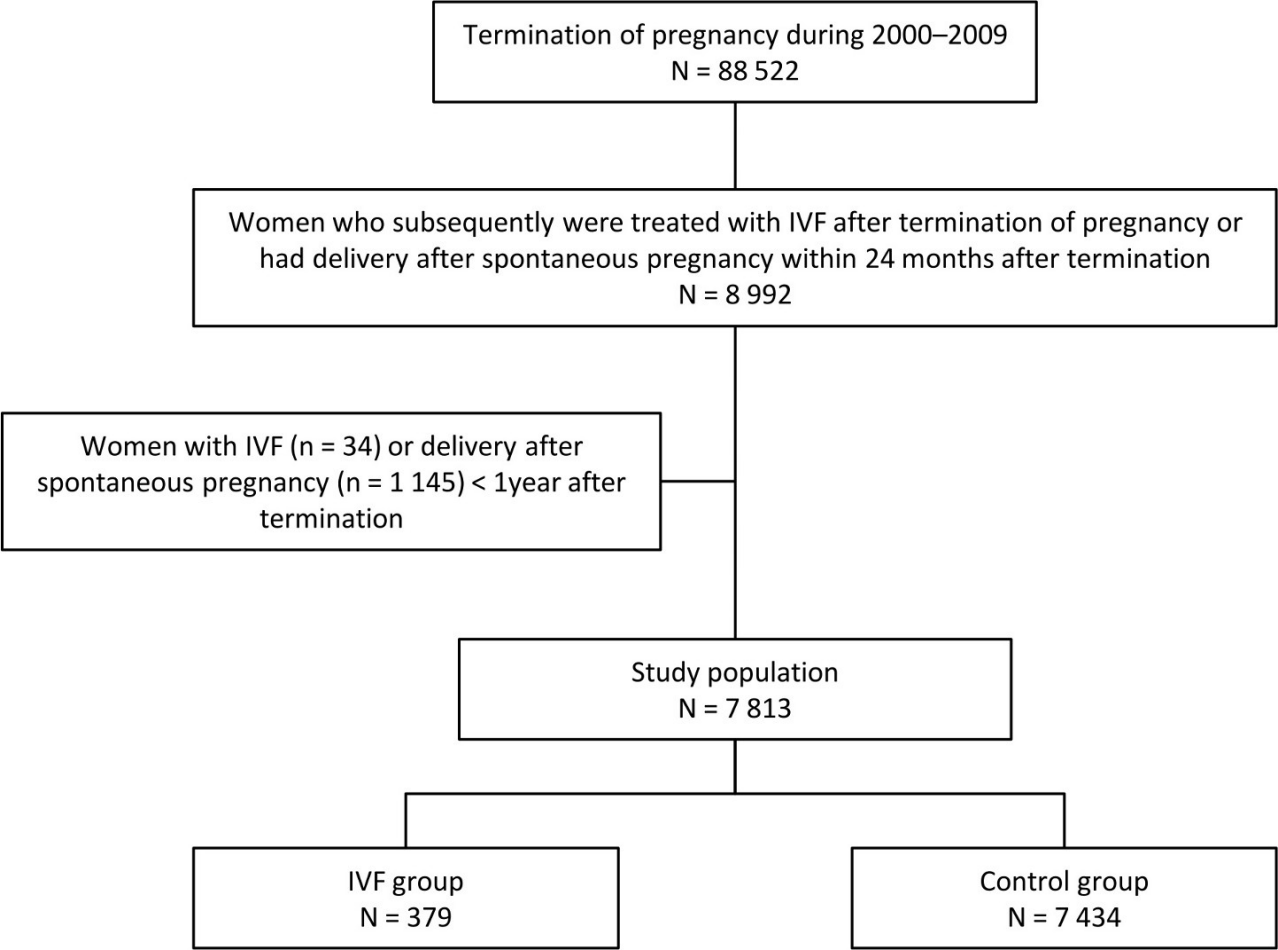
Flow chart detailing the study groups.

In this population-based register study, we identified all women undergoing induced abortion between 2000 and 2009 (n = 88 522) by using the Finnish Register of Induced Abortions (Fig 1). Information on these women was linked to the Drug Reimbursement Register, the Procedures Reimbursement Register, the Hospital Discharge Register, and Medical Birth Register in order to identify women, who subsequently underwent IVF treatment or had a spontaneous pregnancy and delivery 12–24 months after abortion with no infertility drugs, procedures or diagnoses recorded into the registers. Women receiving IVF treatments were identified by searching through registers special infertility drug codes, IVF procedures (Table 1) and diagnoses indicating infertility. If a woman had a registered IVF procedure, or had used combination of gonadotrophin releasing hormone agonist or antagonist and gonadotrophins, or had used gonadotrophin releasing hormone agonist or antagonist and had infertility diagnoses, she was considered as being treated with IVF. Women who had used only follicle stimulating hormone and had no IVF procedures were excluded as they could not be verified as IVF treated.

**Table 1:** Infertility medication and procedure codes used in identification of IVF cases.

Group	Code	IVF case
Infertility medication ATC codes	H01CA02	nafarelin
	L02AE01	buserelin
	H01CC01	ganirelix
	H01CC02	cetrorelix
	G03GA01	choriogonadotrophin
	G03GA02	menotrophin
	G03GA04	urofollitropin
	G03GA05	follitropin alpha
	G03GA06	follitropin beta
	G03GA09	corifollitropin alpha
IVF procedure codes:	TLW10	Fresh embryo transfer
	TLW11	Transmyometrial embryo transfer
	TLW12	Frozen embryo transfer
	TLW14	Embryo transfer after ICSI
	LBF11	Laparoscopy for assisted fertilization
	LFB12	Hysteroscopy for assisted fertilization
	LAA10	Percutaneous or transvaginal puncture and recovery of oocyte
	LAA11	Laparoscopic recovery of oocyte

The induced abortion preceding the IVF treatment or delivery was considered the index abortion. Infertility treatments were identified until the end of 2010 and deliveries until end of 2009. Women who had deliveries between induced abortion and IVF treatment, women who had infertility treatments prior to the index abortion during study period, and women who had used only gonadotrophin releasing hormone agonist or antagonist and had diagnoses indicating endometriosis, were excluded. In the medical literature, trying to conceive without conception for at least one year is referred as infertility or subfertility [12], hence the women who were treated with IVF within less than a year after the abortion (n = 34) were excluded. In order to exclude possible unsuccessful induced abortions leading to continuing pregnancy and delivery, women who delivered less than a year after the abortion (n = 1145) were also excluded.

Women who met the inclusion criterion were divided into two groups: those who had their first IVF procedure at least 12 months after the index induced abortion were chosen as the IVF group (n = 379) and those who had no IVF treatment, but had spontaneous pregnancy and delivery within 12–24 months after the index abortion, were chosen as the comparison group (n = 7434) (Fig 1). By choosing women who deliver soon after the abortion we could eliminate the possibility of subfertility, and could compare groups who did not have difficulties in getting pregnant to those who had such problems. The background characteristics at the time of abortion and the factors associated with the index abortion were compared between the two groups. Furthermore, the use of IVF treatment in the future was evaluated according to different background factors.

The data on background characteristics including age, marital status, occupation, type of residence, as well as number of previous births, miscarriages and induced abortions were retrieved from the Register of Induced Abortion. Socioeconomic status (SES) was defined according to the stated occupation or the highest educational level reported to the Register of Induced Abortion. Coding was based on national standards published by Statistics Finland [13–15]. The factors associated with abortion included gestational age at abortion determined usually by ultrasonographic examination, indication and method of abortion, and complications after abortion, and were retrieved from Register of Induced Abortions and completed with the data on complications obtained from Hospital Discharge Register. Methods of abortion were divided to medication, surgical, and other (e.g. abortion by means of hysterotomy or otherwise specified). Gestational age at abortion was divided to early (≤ 12 weeks) and late (> 12 weeks) according to the Finnish legislation on induced abortions. Complications of the abortion were defined as an infection, hemorrhage, incomplete abortion or surgical re-evacuation of retained products of conception after abortion. In order to identify abortions with complication(s), the diagnoses indicating infection, hemorrhage and incomplete abortion based on ICD-10 and codes for surgical evacuations based on NOMESCO Classification of Surgical Procedures were searched through the data within 42 days after abortion.

The study was approved by the Ethics Committee of the Northern Ostrobothnia Hospital District (no.28/2010). The Finnish Institute for Health and Welfare gave permission (THL/659/5.05.00/2010) to use personal-level data from the national health registers. Personal identification numbers were removed and the data was fully anonymized before all analyses.

### Statistical analyses

Statistical analyses were performed using PASW 23 for Windows (SPSS Inc., Chicago, IL, USA). To assess the differences of the background characteristics and abortion-associated background factors (gestational age at abortion, method of abortion, indication of abortion, complications of abortion) between the study groups Chi-square or Fisher’s exact test were used as appropriate for categorical variables and Mann-Whitney U-test for continuous variables. Bivariable logistic regression was used to assess whether potential covariates (i.e. the background characteristics or the abortion associated background factors) increased the use of IVF treatment compared with that in comparison women. Possible multicollinearity among the potential covariates was examined by calculating the Variance Inflation Factor (VIF). Age at the time of induced abortion, socioeconomic status, number of previous abortions and number of previous deliveries were chosen as covariates in the final multivariable logistic regression analysis, as these factors were found to be relevant confounders and also showed association in bivariable analysis. The level of statistical significance was set at p < 0.05.

## Results

Of the 88 522 women who underwent induced abortion, 379 (0.4%) received subsequent IVF treatment. Women treated by means of IVF were older, of higher socioeconomic status and had fewer previous induced abortions and deliveries compared with women in the comparison group (Table 2). The median age at the time of IVF treatment was 32 years (IQR 28–37) and the mean time between abortion and IVF treatment was 4.7 years (range 1.1–10.6). No significant differences were observed between the IVF and comparison groups in the abortion-associated factors: gestational age at abortion (≤ 12 weeks or > 12 weeks), method of abortion or complications at abortion. The median of gestational age at the time of abortion was lower in IVF group (p = 0.006) (Table 3).

**Table 2:** Demographic characteristics of the women at the time of induced abortion. Data are reported as number of women (%) unless stated otherwise.

Characteristics	IVF group (n = 379)	Comparison group (n = 7434)	P
Age, years:			
Median (IQR)	28 (23–33)	25 (20–30)	< 0.001
≤ 19	44 (11.6)	1490 (20.0)	< 0.001
20–24	70 (18.5)	2197 (29.6)	
25–29	103 (27.2)	1768 (23.8)	
30–34	95 (25.1)	1256 (16.9)	
35–39	52 (13.7)	609 (8.2)	
≥ 40	15 (4.0)	114 (1.5)	
Marital status:			0.172
Married	75 (19.8)	1628 (21.9)	
Cohabiting	73 (19.3)	1539 (20.7)	
Single	200 (52.8)	3767 (50.7)	
Divorced or widowed	30 (7.9)	498 (6.7)	
Unknown	1 (0.3)	2 (0.0)	
Socioeconomic status:			< 0.001
Upper white-collar worker	67 (17.7)	603 (8.1)	
Lower white-collar worker	110 (29.0)	1700 (22.9)	
Blue-collar worker	45 (11.9)	1209 (16.3)	
Students	87 (23.0)	1919 (25.8)	
Other	17 (4.5)	690 (9.3)	
Unknown	53 (14.0)	1313 (17.7)	
Type of residence:			0.151
Urban	296 (78.1)	5418 (72.9)	
Densely populated	39 (10.3)	986 (13.3)	
Rural	44 (11.6)	1026 (13.8)	
Unknown	0 (0.0)	4 (0.1)	
Number of previous abortions:			0.001
0	295 (77.8)	5114 (68.8)	
1–2	75 (19.8)	2139 (28.8)	
≥ 3	9 (2.4)	180 (2.4)	
Unknown	0 (0.0)	1 (0.0)	
Number of previous deliveries:			< 0.001
0	266 (70.2)	3718 (50.0)	
1–2	92 (24.3)	3071 (41.3)	
≥ 3	21 (5.5)	645 (8.7)	
Number of previous miscarriages:			0.663
0	319 (84.2)	6204 (83.5)	
1–2	55 (14.5)	1154 (15.5)	
≥ 3	5 (1.3)	76 (1.0)	

IQR = Inter-quartile range, IVF = In vitro fertilization.

**Table 3:** Induced abortion-associated background factors. Data are number of women (%) unless stated otherwise.

Characteristics	IVF group (n = 379)	Comparison group (n = 7434)	P-value
Gestational age at abortion (wk):			
Median (IQR)	8 (7–11)	9 (7–11)	0.006
≤ 12	324 (85.5)	6217 (83.6)	0.339
> 12	55 (14.5)	1217 (16.4)	
Method of abortion:			0.187
Medication	190 (50.1)	4046 (54.4)	
Surgical	189 (49.9)	3386 (45.5)	
Other	0 (0.0)	2 (0.0)	
Indication for abortion:			0.198
Social	314 (82.8)	6231 (83.8)	
Age < 17 y	8 (2.1)	205 (2.8)	
Age ≥ 40 y	5 (1.3)	32 (0.4)	
≥ 4 children	3 (0.8)	83 (1.1)	
Fetal indication	49 (12.9)	858 (11.5)	
Other	0 (0.0)	25 (0.3)	
Surgical re-evacuation after abortion:			0.808
Yes	37 (9.8)	698 (9.4)	
No	342 (90.2)	6736 (90.6)	
Haemorrhage after abortion:			0.568
Yes	31 (8.2)	672 (9.0)	
No	348 (91.8)	6762 (91.0)	
Incomplete abortion:			0.239
Yes	21 (5.5)	318 (4.3)	
No	358 (94.5)	7116 (95.7)	
Infection after abortion:			0.625
Yes	13 (3.4)	292 (3.9)	
No	366 (96.6)	7142 (96.1)	

IQR = Inter-quartile range, IVF = In vitro fertilization.

**Fig 2:**
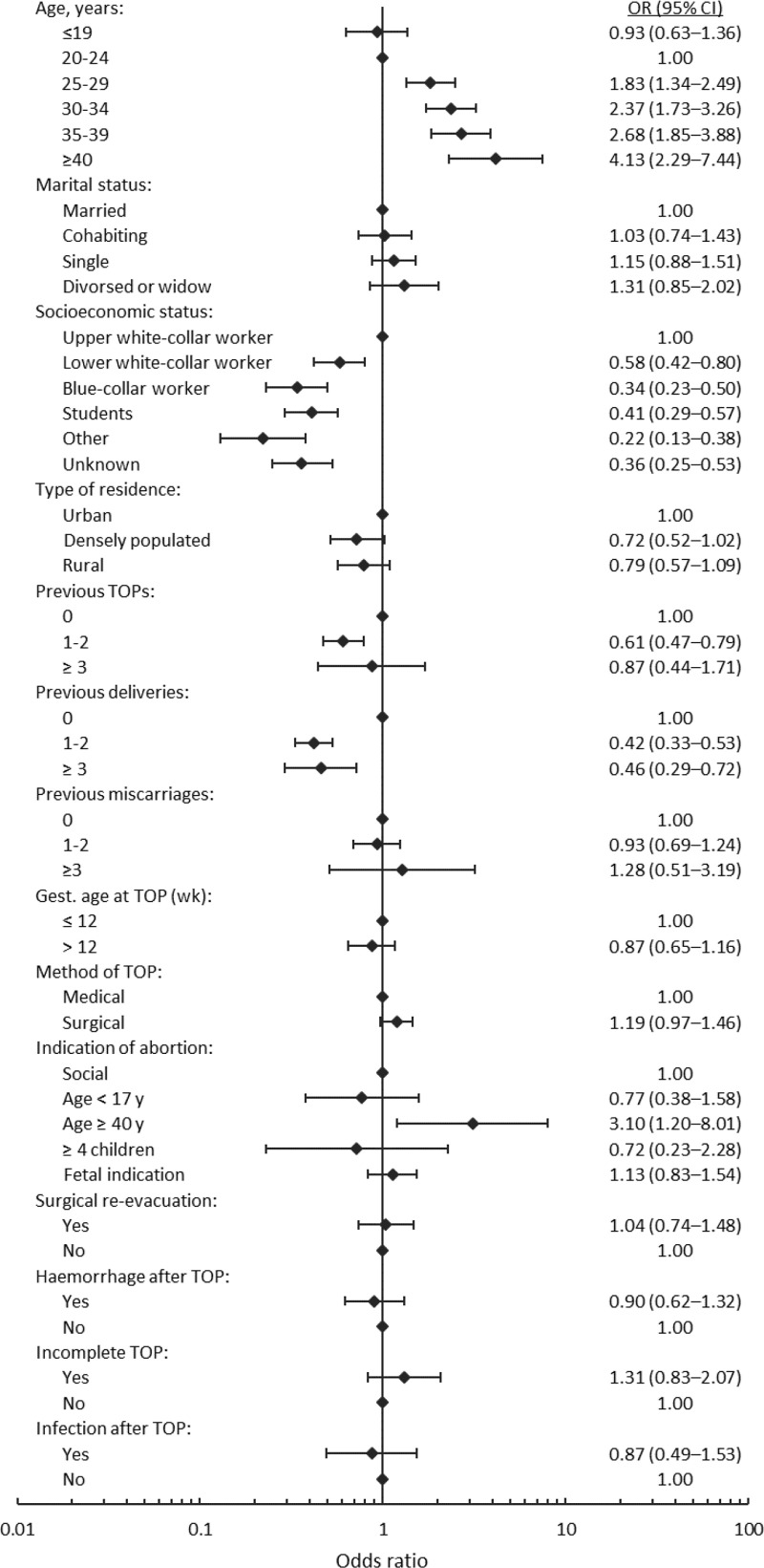
The association of characteristics, and induced abortion-associated background factors, with the future IVF treatment (unadjusted odds ratios) (n = 7813). Note the logarithmic scale on the horizontal axis. TOP = Termination of pregnancy.

In bivariable analysis the use of IVF treatment increased with age at the time of induced abortion (Fig 2), and was highest among women aged 40 or more (OR 4.13, 95% CI 2.29–7.44). Greater parity and lower socioeconomic status at the time of abortion were associated with lower use of IVF treatment. History of previous induced abortion at the time of index abortion also lowered the risk of using IVF treatment when compared to women with no such history. The association vanished for women with three or more abortions. Gestational age at abortion, the method of abortion and complications at the time of abortion, such as surgical re-evacuation, haemorrhage, incomplete abortion or infection, were not associated with the future IVF treatment. When the indication for induced abortion was age above 40 years, the use of IVF was increased.

**Fig 3:**
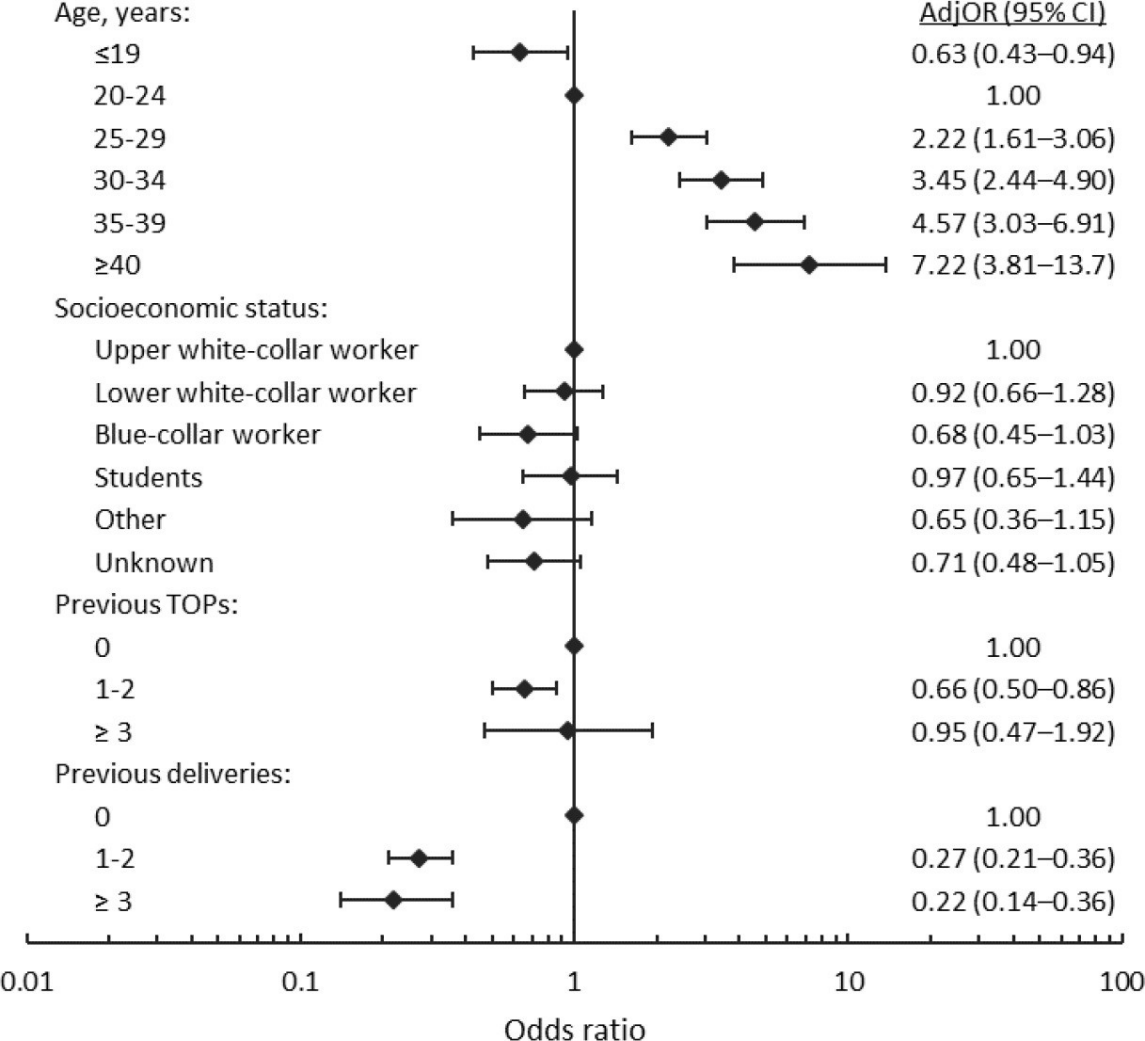
The association of characteristics, and induced abortion-associated background factors, with the future IVF treatment (adjusted odds ratios) (n = 7813). Note the logarithmic scale on the horizontal axis. TOP = Termination of pregnancy.

In multivariable analysis after adjustments, higher age was still associated with the use of IVF treatment. Women with previous deliveries or with a history of one or two induced abortions had lower use of IVF. The association between socioeconomic status and IVF treatment was no longer statistically significant (Fig 3). When the indication for termination was age of 40 or above, the use of IVF treatment was increased in the bivariable analysis, but this variable was not included in the multivariable analysis to avoid multicollinearity.

## Discussion

We find that the proportion of women using IVF treatment within 1 to 10 years after induced abortion was low, 0.4%. Moreover, factors associated with induced abortion such as gestational age at abortion, method of abortion and complications following abortion were not associated with the use of IVF treatment later. Instead, the factors associated with IVF were those generally known to associate with infertility: advanced age and a lower number of previous deliveries at the time of induced abortion. The number of previous abortions was lower in the IVF group and a history of abortion was associated with lower use of IVF, which supports the conclusion that IVF was mainly due to other infertility factors and not related to the induced abortion.

This study is based on high-quality health registers. In a recent study, the coverage of the Finnish Register of Induced Abortions was shown to be high, 97% of induced abortions were reported in the register and over 90% of individual variables were exactly matched in clinical and register data in a recent study [16]. Another study showed that identification of infertility treatments based on treatment reimbursements is effective [17].

One of the limitations of our study is the fact that apart from IVF, other infertility treatments such as induction of ovulation and intrauterine insemination could not be reliably identified by using these registers. Furthermore, we could not comprehensively differentiate women who were treated with IVF because of male factor infertility or women with miscarriages after induced abortion because of lack of appropriate registration. However, as the potential mechanism behind abortion-related infertility is the damage of the fallopian tubes, endometrium, or mechanical trauma, lacking information of ovulation induction, intrauterine insemination and male factor infertility will probably not affect the results. We followed deliveries only until the end of 2009 and the use of IVF-treatments until 2010. However, the potential bias caused by this would overestimate the need of IVF after induced abortion. Furthermore, medication abortion became first available in Finland in 2000, and the transition to its dominant use occurred during the following 10 years (approx. 85% in 2009 [5]). Therefore, to be able to assess the effects of surgical *vs*. medication abortion on the use of IVF-treatment, a time period during which the use of the two means of induced abortion was roughly similar was selected. Finally, we only held information on actual IVF treatments performed, but not the need for such treatments. The need of IVF after abortion may be very low because very few of the women undergoing induced abortion reached the age or place in their lives when fertility was a concern. Moreover, the findings presented in this study are applicable in countries where induced abortion is legal and infertility treatments are easily available.

By deciding to terminate the pregnancy, women may postpone their childbearing possibly to an age when natural fertility is in decline. This is the most likely explanation for the future use of IVF treatments after abortion. The mean monthly probability of conception declines progressively after the age of 31 years [18]. The prevalence of one year of infertility increase to more than 30% in the 35–44 -year age group compared to 6% for women less than 24 years-of-age [18]. In our study the results reflect the same influence of age on fertility, when the age at termination was 25 years or more the probability of being treated by means of IVF started to increase and was highest when the age was above 40 years.

In accordance with our study, it has been shown that women who are treated with IVF after induced abortion are more often nulliparous and have fewer previous abortions [19]. Thus, even though the women seeking induced abortion are fertile, it seems that those women who are later treated with IVF might in the first place present more subfertile population of women.

Damage to the fallopian tubes after induced abortion due to infection or mechanical trauma could theoretically lead to tubal infertility and IVF treatments. In our study, the abortion-related complications, such as infection or surgical re-evacuation did not associate with IVF treatment in the future. Two previous case-control studies have not found an association between a positive history of induced abortion and tubal pathology [20, 21]. Furthermore, even though the risks of incomplete abortion and surgical re-evacuation are higher after medication, compared to surgical induced abortion [22], the medication method of abortion was not associated with the future IVF treatments in our study.

There are no studies evaluating the impact of medication induced abortion on future fertility. However, a study comparing the natural conception rates of women after medication (n = 131) and surgical (n = 130) treatment of miscarriage has shown that the natural conception rates after both methods are 98% and the cumulative pregnancy rates are similar [23]. According to the present study prior medication abortion does not result in subsequent infertility requiring IVF.

Based on the results of this large study, the overall future use of IVF treatment after abortion is low. One explanation for this low use of IVF is the fact that the women having unplanned pregnancies and undergoing induced abortion are generally highly fertile. The use of IVF is associated with higher age and a lower overall number of deliveries and induced abortions at the time of induced abortion, rather than abortion-associated factors. The factors found to be associated with a future IVF are those generally recognized as risk factors of infertility. These reassuring data are important when counseling women requesting an induced abortion.
